# Lesions of the dorsomedial striatum delay spatial learning and render cue-based navigation inflexible in a water maze task in mice

**DOI:** 10.3389/fnbeh.2014.00042

**Published:** 2014-02-13

**Authors:** Anni S. Lee, Jessica M. André, Christopher Pittenger

**Affiliations:** ^1^Department of Psychiatry, Yale UniversityNew Haven, CT, USA; ^2^Weill Cornell Graduate SchoolNew York, NY, USA; ^3^Department of Psychology, Yale UniversityNew Haven, CT, USA; ^4^The Child Study Center, Yale UniversityNew Haven, CT, USA

**Keywords:** striatum, basal ganglia, learning, habit, mouse, behavioral flexibility

## Abstract

The dorsal striatum is involved in cue-based navigation strategies and in the development of habits. It has been proposed that striatum-dependent cued navigation competes with hippocampus-dependent spatial navigation in some circumstances. We have previously shown that large lesions of the dorsal striatum, as well as impairment of corticostriatal synaptic plasticity in transgenic mice, can enhance spatial learning in a water maze task, presumably by the disruption of competitive interference. However, the dorsal striatum is not a homogeneous structure; both anatomical considerations and experimental studies in various paradigms show that dorsomedial and dorsolateral striatum are functionally distinct, although there is no precise anatomical or neurochemical boundary between them. Here we investigated the effect of restricted excitotoxic lesions of dorsomedial striatum (DMS) on cued and spatial water maze learning. We find that dorsomedial striatal lesions delay spatial learning but permit cued learning. After cued learning, lesioned animals showed inflexible search, resulting in repeated visits to the escape platform-associated cue. These results support a role for the DMS in behavioral flexibility rather than in cue-based navigation.

## Introduction

The dorsal striatum participates in the formation of procedural memories (Graybiel, 1998, 2008; Packard and Knowlton, 2002; Yin and Knowlton, 2006). In humans, the dorsal striatum (the caudate and putamen) is implicated in cue-based navigation (Hartley et al., 2003), implicit pattern recognition and classification (Knowlton et al., 1996; Poldrack et al., 2001) and artificial language acquisition and fluency (Forkstam et al., 2006). Neuropsychiatric conditions that affect the striatum can impair these behaviors without affecting explicit memory (Knowlton et al., 1996; Deckersbach et al., 2002; Marsh et al., 2004). In rats, the dorsal striatum is implicated in cue-based navigation (Packard et al., 1989; Jog et al., 1999; Packard and McGaugh, 1992), egocentric navigation (Packard and McGaugh, 1996), and instrumental habit (Yin et al., 2004). We and others have extended this work to mice (Pittenger et al., 2006; Lee et al., 2008; Quinn et al., 2013).

The multiple memory systems hypothesis proposes that interacting systems in the brain work in parallel to discern regularities in the environment and use them to guide behavior (Tolman, 1949; White and McDonald, 2002). When memory systems operate in parallel they can interact, and these interactions can sometimes be competitive (Poldrack and Packard, 2003; Daw et al., 2005). Competition between memory systems is supported by brain imaging studies in humans, in which striatal and hippocampal activation are inversely correlated during task performance (Poldrack et al., 2001). In rodents, lesions of the hippocampus can enhance striatum-dependent cued learning (Packard et al., 1989; Lee et al., 2008), and dorsal striatal dysfunction can enhance spatial learning (Lee et al., 2008).

However, the dorsal striatum is not a homogeneous structure. It is commonly subdivided into medial and lateral compartments, roughly homologous to the primate caudate nucleus and the putamen. Dorsomedial striatum (DMS) receives input primarily from association neocortex and is therefore likely to process multimodal and cognitive information. In contrast, dorsolateral striatum receives input from primary sensory and motor cortices and is therefore more likely to be involved in direct sensory-motor associations such as habits (Gerfen, 2004; Yin, 2010). While disruption of dorsolateral striatal function disrupts cued learning and habit, more restricted disruption of DMS impairs spatial learning and leaves cued learning intact (Devan and White, 1999; Yin and Knowlton, 2004; Yin et al., 2004, 2005a,b; McDonald et al., 2008; Moussa et al., 2011; Quinn et al., 2013). The DMS is thought to contribute to behavioral flexibility and reversal learning (Ragozzino, 2007; McDonald et al., 2008; Castane et al., 2010).

We sought to address two limitations to these findings. First, most studies examining the differential function of striatal subregions have been performed in rats; while there are a few recent exceptions to this pattern (Yin et al., 2009; Quinn et al., 2013), none have specifically addressed the issue of behavioral flexibility in a navigational task after DMS damage. Since mice are increasingly used as a model system for molecular and cell type-specific studies, it is critical to demonstrate that this functional differentiation within the basal ganglia is conserved. Second, examination of cued learning is subject to numerous performance-related confounds (coordination, vision, and so forth); a recently developed water maze task (Lee et al., 2008) controls for these optimally by assaying cued and spatial learning in parallel in procedurally identical tasks.

In this context, we hypothesized that restricted dorsomedial striatal lesions in mice would impair behavioral flexibility and spatial learning in mice in our cued and spatial water maze task. Both hypotheses were confirmed; lesioned animals showed delayed learning in a spatial water maze task, whereas in the cued water maze task, dorsomedial lesions did not impair learning. However, in a probe trial, lesioned animals exhibited perseverative return to the goal, after control animals had switched to a broader pattern of search. Taken together, these data are consistent with a role for the DMS in behavioral flexibility. Dysfunction of this region may contribute to inflexible patterns of behavior or thought in neuropsychiatric disease.

## Methods

### Animals

All experiments were conducted under the supervision of Yale University’s Institutional Animal Care and Use Committee (Animal Welfare Assurance Number A3230-1). Food (standard laboratory chow) and water were available *ad libitum*. All experiments examined adult male C57Bl/6 mice acquired from Jackson Laboratories,^1^ 2.5–6 months of age.

### Surgery

Stereotaxic surgery was performed on 2.5–3.0 month old male mice, following standard procedures, under sterile conditions. Anesthesia was by intraperitoneal injection of tribromoethanol (TBE: Sigma) dissolved in 2-methyl-2-butanol (Sigma) and then diluted 1:40 in normal saline (total dose 275 mg TBE/kg). Sterile lubricant was generously applied to the eyes. The scalp was incised and the skin retracted. Bregma and lambda were leveled in the dorsoventral plane. Bilateral burr holes were drilled through the skull at the target anterior-posterior and medial-lateral coordinates, as measured from bregma. A 0.5 µl Hamilton syringe was lowered into each of these holes in turn.

**Figure 1:**
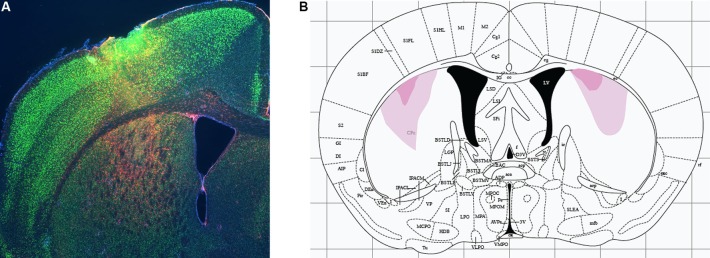
**Excitotoxic lesions of dorsomedial striatum. (A)** Excitotoxic lesions were produced by stereotaxic infusion of NMDA; control animals received equivalent infusions of saline. Lesions were documented by immunohistochemistry for glial fibrillary acidic protein (GFAP, documenting region of gliosis and therefore of neuronal damage; red) and NeuN (marking healthy neurons). A sample DMS lesion (from a lesion refining experiment, not from an animal used in behavioral analysis) is shown. **(B)** Minimum and maximum lesion extent of animals whose behavior was analyzed.

Excitotoxic lesions were performed by manual infusion of 0.1 µl NMDA (Sigma; 20 mg/ml in sterile saline) over the course of 1 min. Targeting coordinates were determined from Paxinos and Franklin (2004) and refined empirically in pilot experiments to achieve targeting of the DMS without damage to dorsolateral striatum (with these subregions defined by analogy to rat as per Gerfen, 2004). Infusion coordinates were AP 0.74 mm, ML ±2.3 mm, DV −3.5 mm. These coordinates are identical to those used for larger lesions in a previous study (Lee et al., 2008), but the smaller volume of NMDA was found in pilot experiments to lead to substantially more restricted lesions than in that previous study, as illustrated in Figure 1. Sham animals received identical infusions of sterile saline. The syringe remained in place for 4 min to allow for diffusion of the drug.

Following the second infusion, the incision was sutured and a topical antibiotic ointment applied. Mice were allowed to recover on a heat pad until they recovered from anesthesia. Upon awakening they were returned to their home cage and allowed to recover for at least 14 days prior to the start of behavioral testing.

### Behavioral testing

Water maze training and testing were performed as previously described (Lee et al., 2008). Briefly, animals learned to escape a circular pool of opaque water by swimming to one of two visually distinct cues. The pool was 164 cm in diameter; an escape platform was present during training, 12 cm square, located 1 cm below the surface of the water and therefore invisible to the animal. Three distinct visible cues were used; cues consisted of plastic cylinders, 11 cm high × 2.5 cm diameter, painted either uniform gray or with sharp black-and-white stripes, 1 cm in width, oriented either horizontally or vertically.

The first 5 days consisted of shaping to the task. On day 0 animals were placed on the platform four times (20 min inter-trial interval). On days 1 through 4, the escape platform was marked with the uniform gray cue; animals were placed in the pool and allowed 120 s to swim to it.

Following shaping, animals were trained in the two-cue task for 7 days; each animal was trained in either the cued or spatial task, never in both. All experiments consisted of four trials per day, with a 20 min inter-trial interval. In the **cued task** the escape platform was moved on each trial but was reliably marked by one of the two cues (i.e., either horizontal or vertical stripes, held constant throughout training for each animal but counterbalanced across animals within each group). In the **spatial task** the escape platform was always in the same location but was pseudorandomly associated with the striped cues. In both tasks the second visible cue (the **lure**) was present in a quadrant adjacent to the escape platform and its associated cue (the **goal**) on a stand that held it at an identical height in the water but did not permit escape. Latency to find the escape platform was measured for all training trials; search was recorded by an overhead digital camera.

Learning was assayed using a **probe trial**, administered in place of the fourth training trial after 3, 5, and/or 7 days of training, as specified below for each experiment. In the probe trial both goal and lure cues were placed on stands that did not allow escape; the animal’s search was monitored by an overhead camera over 60 s. Extra-maze cues were identical to those present in a training trial. In both the cued and the spatial task, a systematic bias towards the goal cue relative to the lure cue (that is, towards the location where the platform would have been on a regular training trial) was interpreted as evidence of learning. This was quantified by quadrant occupancy. Other measures (mean distance from the goal and lure cues during search and occupancy in circular zones centered on the goal and lure cues) gave similar results (not shown). Probe trial track analysis was performed using Ethovision® (Noldus, Leesburg, VA).

### Documentation of lesions

Excitotoxic lesions were documented using immunohistochemistry for glial fibrillary acidic protein (GFAP) and NeuN, to label glia and neurons, respectively. Nissl staining gave similar results but documented lesions less clearly in striatum than GFAP staining. Mice were euthanized by cervical dislocation and their brains rapidly dissected and fixed overnight in freshly prepared 4% paraformaldehyde/PBS at 4°C. After fixation, brains were equilibrated with 30% sucrose and sliced on a microtome at 40 µm; floating sections were stored in a cryoprotectant solution (30% glycerin, 30% ethylene glycol, 0.2X PBS) at 4°C. Sections were washed 3 × 10 min in 1X PBS, blocked with PBS/0.3% Triton/2% goat serum (Sigma) for an hour with gentle shaking at room temperature, and then immunostained for GFAP (Sigma rabbit polyclonal anti-GFAP IgG, G9269, 1:500) and NeuN (Chemicon International mouse monoclonal anti-NeuN IgG, MAB377, 1:1000) in PBS/0.3% Triton. The following day, slices were rinsed twice in PBS/0.3% Triton and twice in PBS, stained for 1 h with secondary antibodies (Life Technologies: Alexa Fluor 488 goat anti-mouse IgG 1:400; Alexa Fluor 549 goat anti-rabbit IgG 1:400) in PBS/0.3% Triton/2% goat serum, washed again 3X in 1X PBS, and mounted on glass slides. GFAP and NeuN immunoreactivity were visualized on an upright Nikon fluorescent microscope.

### Statistical analysis

All data were organized using Microsoft Excel and analyzed using SPSS (IBM). All data met assumptions of normality for parametric statistics. Latency data were analyzed by RM-ANOVA, with group (NMDA vs. saline) as a between-subject factor and day and trial as nested within-subject factors, as in Lee et al. (2008). Probe trial data were analyzed by ANOVA, with group (NMDA vs. saline) as a between-subject factor and cue (goal vs. lure) and probe trial day as within-subject factors.

## Results

### Dorsomedial striatal lesions do not affect swimming or other aspects of task performance

We targeted lesions to the DMS, defined anatomically with reference to Gerfen (2004). Lesions were produced by infusion of NMDA, as in our previous studies (Lee et al., 2008; Baldan Ramsey et al., 2011; Quinn et al., 2013); control animals received equivalent infusions of sterile saline. Following behavioral analysis, animals were sacrificed and lesion location and extent were documented by immunohistochemistry to GFAP and NeuN (see Methods); a sample lesion is shown in Figure 1A. Minimal and maximal lesion extents of the animals included in behavioral analysis are shown in Figure 1. Resolution in the A-P dimension was limited by sampling density (we examined coronal sections every 200–300 µm); lesions appeared to be symmetrical in the A-P and M-L dimensions, suggesting an A-P extent at the widest point of 250–500 µm from the section illustrated.

**Figure 2:**
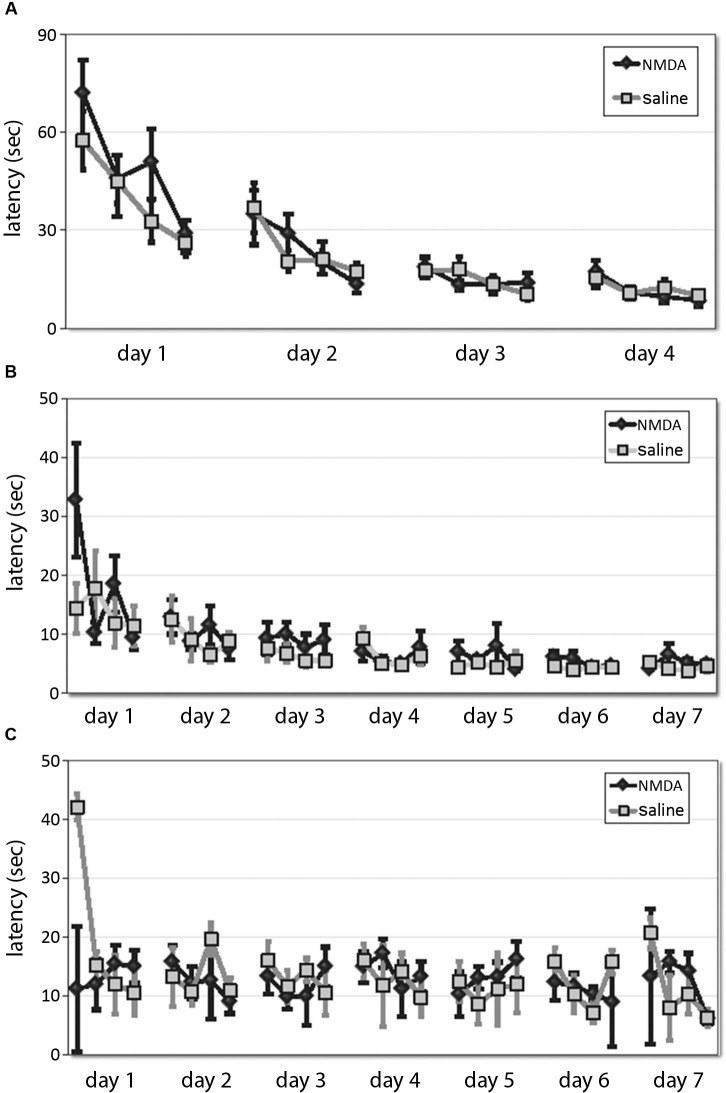
**DMS lesions do not affect animals’ latencies in shaping, spatial, or cued task. (A)** Latencies of lesioned and control mice did not differ over 4 days of training to locate a platform marked by a single visible cue in the shaping phase of the water maze task. **(B)** In the subsequent 7 days of training in the 2-cue spatial or cued task, latencies decreased across training and were lower in the spatial task, but there was no effect of lesion either overall. **(C)** Similarly, in the cued task there was no effect of lesion on latency.

Animals were trained in either the spatial or the cued version of the 2-cue water maze task (Lee et al., 2008; see Section Methods). The first phase consists of shaping to the task, in which animals learn to swim to a single cue of variable position. DMS lesions did not affect animals’ ability to learn this task, as shown by equivalent latency curves over 4 days of training (RM-ANOVA: main effect of day, *F*_[3,31]_ = 43.17, *p* < 0.001; main effect of trial, *F*_[3,31]_ = 23.06, *p* < 0.0001; day × trial interaction, *F*_[9,25]_ = 4.124, *p* = 0.002; effect of lesion and interactions NS (Figure 2A)). One animal (a control) never learned the one-cue task and developed difficulty swimming; it was excluded from subsequent testing and analysis.

Animals were then trained in either the spatial (*n* = 8 lesioned, 8 control) or cued task (*n* = 9 lesioned, 9 control; all *n* are after exclusions) across 7 days. Latencies decreased across training (RM-ANOVA: main effect of day, *F*_[6,180]_ = 10.44, *p* < 0.001; main effect of trial, *F*_[3,90]_ = 8.209, *p* < 0.001; day × trial interaction, *F*_[18,540]_ = 1.79, *p* < 0.05) and were lower in the spatial task (RM-ANOVA: main effect of task, *F*_[1,30]_ = 15.44, *p* < 0.001), but there was no effect of lesion in either overall (main effect of lesion and interactions NS). While the latencies in the spatial task (Figure 2B) were significantly shorter than those in the cued task (Figure 2C), as in previous work (Lee et al., 2008, supplementary data), there was no effect of lesion on latency (spatial: RM-ANOVA: main effect of lesion, *F*_[1,14]_ = 3.23; *p* > 0.05; lesion × day and lesion × trial effects, *p* > 0.2, cued: RM-ANOVA: main effect of lesion, *F*_[1,16]_ = 0.127, *p* > 0.5), although there was a trend-level lesion × trial interaction (*F*_[3,14]_ = 3.12, *p* = 0.06). This suggests that these striatal lesions did not impair animals’ ability to see or to swim, their motivation to escape the water, or other procedural aspects of the water maze task.

### Dorsomedial striatal lesions retard spatial learning

**Figure 3:**
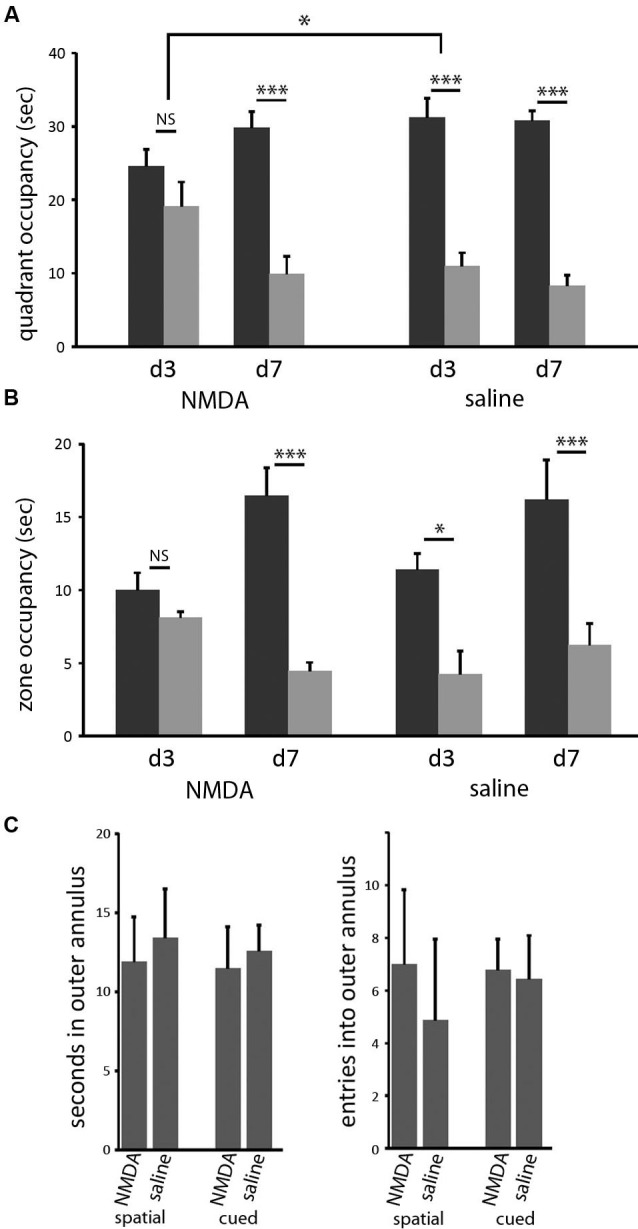
**DMS lesions delay spatial learning without affecting asymptotic performance. (A)** Control mice (*n* = 8) had acquired the spatial task by the end of the third day of training, as indicated by their bias towards the goal cue quadrant in the probe (*p* < 0.005); in contrast, mice with dorsomedial striatal lesions (*n* = 8) did not show a significant bias towards the goal quadrant. This effect of lesion was significant (*p* < 0.05). In contrast, both groups showed clear spatial learning on day 7 (both: *p* < 0.005), with no significant difference between groups. Similar effects were seen when probe trial data were analyzed by goal zone occupancy or proximity (not shown). **(B)** The same pattern was seen when probe trial performance was quantified by occupancy in a circular target zone around the goal and lure cues. **(C)** Lesions did not cause any increased thigmotaxis in either task on day 3. There was no significant effect of either lesion or task on either occupancy in or entries into the outer annulus during exploration. * *p* < 0.05; *** *p* < 0.005.

In both the classic spatial Morris water maze task and this 2-cue water maze task, a more sensitive measure of learning (as opposed to other task-relevant capacities) is provided by a probe trial. We performed probe trials on the last trial of days 3 and 7 of training, as previously described (Lee et al., 2008; see Methods). On the day 3 probe trial, control animals showed robust spatial learning (paired *t-*test of goal vs. lure quadrant: *t* = 4.676; *p* < 0.005), as we have seen previously, but lesioned animals showed only a nominal spatial bias in their search, which did not reach significance (*t* = 1.052;* p* > 0.3). In the between-group comparison, the effect of lesion was significant (ANOVA: quadrant × lesion interaction, *F*_[1,14]_ = 4.828, *p* < 0.05). Both groups showed spatial bias by day 7 probe trial (both: *p* < 0.005), with no significant difference between groups (ANOVA: quadrant × lesion interaction, *p* > 0.5 both days; Figure 3). Therefore, in this water maze task in mice, as has been seen in rat in a different context (Devan and White, 1999), restricted DMS lesions retard spatial learning without affecting asymptotic performance.

Similar effects were seen when probe trial data were analyzed in other ways. We measured the time during the probe trials that each animal spent inside circles (40 cm diameter) centered on the goal and lure cues. In pilot experiments (not shown) we have found this method of quantification to be slightly less sensitive to learning in the spatial task, in which search is broader within the target quadrant, but significantly more sensitive to learning in the cued task, in which search tends to be very tightly focused on the visible cue. Again, control animals showed clear spatial learning after 3 days of training, while lesioned animals did not develop significant spatial bias until day 7 (Figure 3B). When these probe trials were broken up into 15 s blocks, there was no difference in spatial bias across the course of the probe trials for any trial in either group (not shown).

It has been suggested that such a disruption of spatial learning may derive from navigational inefficiency, as manifested by an increase in thigmotaxis early in training (Devan et al., 1999). We therefore examined thigmotaxis in our animals at the time of the day 3 probe trial (at which time a significant effect of lesion on spatial performance is observed). Thigmotaxis was defined as time spent within 10 cm of the pool wall, a zone that comprises 23% of the total are of the pool.We found no effect of lesion (or of task) in thigmotaxis (2 × 2 ANOVA: no main effects or interactions). Entries into this outer annulus were also quantified. This gives another measure of search bias with respect to the pool wall; there was again no effect of lesion or task (Figure 3C).

### Dorsomedial striatal lesions do not impair cued learning but render search inflexible

**Figure 4:**
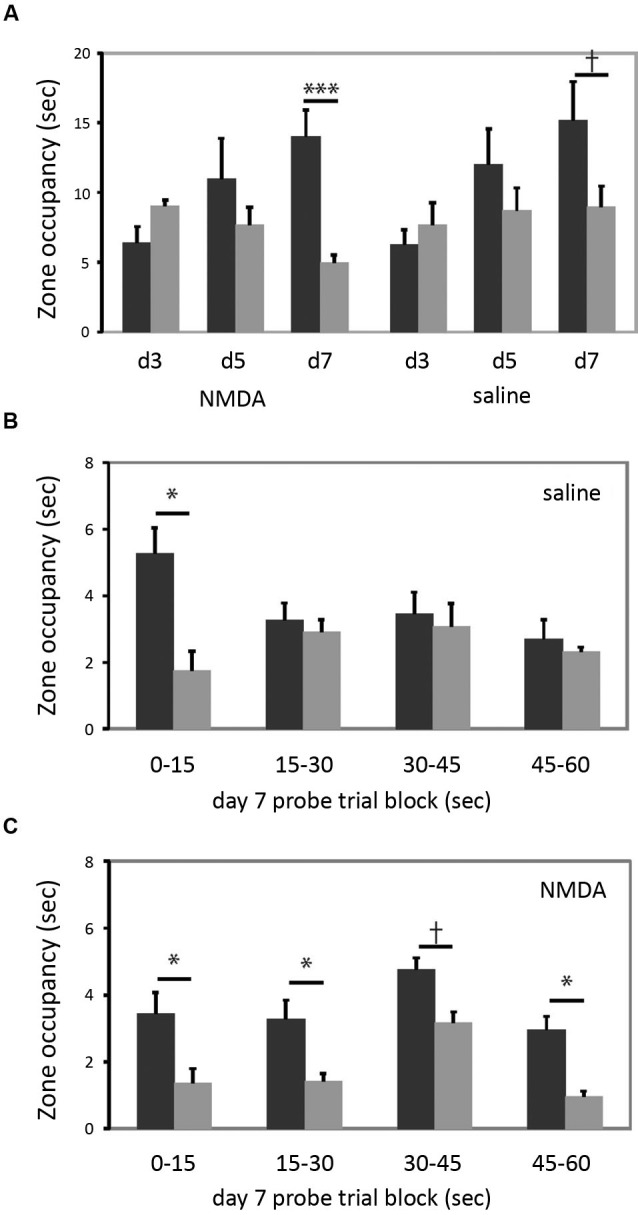
**DMS lesions do not impair cued learning. (A)** Both lesioned and control mice (*n* = 9 in each group) developed a bias towards the reinforced cue by the day 7 probe trial. When groups were analyzed separately for day 7 target zone occupancy, there was a highly significant difference in target zone occupancy in lesioned animals; the bias was present at trend level in control animals. **(B)** This quadrant bias was apparently only in the first 15 s of search in control animals; for the balance of the probe trial they searched more broadly. Quadrant bias was significant during the first 15 s (*p* < 0.05) but non-significant during all other blocks (all *p* > 0.25). **(C)** In contrast, animals with DMS lesions showed a persistent goal-quadrant bias during all probe trial blocks. When examined separately, this bias was significant for blocks 1, 2, and 4, and at trend level for block 3. † *p* < 0.1; * *p* < 0.05; *** *p* < 0.005.

In the cued task, neither group learned by the day 3 probe, but both groups showed a bias towards the reinforced cue on the day 7 probe trial (Figure 4A). The increase in quadrant bias was significant across trials (RM-ANOVA, day × zone interaction, *F*_[2,15]_ = 5.07, *p* < 0.05). There was no effect of lesion either in ANOVA analysis or individual comparisons. Therefore, in striking contrast to larger striatal lesions (Lee et al., 2008), restricted lesions of the DMS do not impair cued learning.

We were struck by the fact that the lesioned animals showed a stronger goal-zone bias than controls in the day 7 probe trial (lesioned mice one-tailed *t-*test for predicted effect: *t* = 4.607, *p* = 0.001; control mice: *t* = 1.597; *p* = 0.075). While this effect of lesion did not reach statistical significance in a between-group contrast, it motivated us to examine the probe trial more closely, in an exploratory analysis.

The probe trial consists of a 60 s search (see Methods); but nearly all animals visit the previously reinforced cue within the first 15 s (all but one of the 34 animals in this experiment did so). We reasoned that search bias towards the reinforced cue should be maximal in the first 15 s. An animal might thereafter switch search strategies, investigating the other cue and the broader pool after finding that the previously reinforced cue did not permit escape. To examine this hypothesis, and whether such a pattern differed between lesioned and control animals, we broke the day 7 probe trial down into 15 s blocks.

As shown in Figure 4B, control animals trained in the cued task exhibited the predicted shift in search pattern over the course of the 60 s probe trial. In the first 15 s there was a marked bias towards the previously reinforced cue (one-tailed *t-*test for predicted effect: *t* = 2.128, *p* = 0.03). However, in each subsequent 15 s block, there was no systematic search bias (all *t* < 1, all *p* > 0.25). This change over the course of the probe trial was manifested by a statistically significant block × zone interaction (RM-ANOVA of control, cued-trained mice: main effect of block, *F*_[3,6]_ = 3.34, *p* = 0.097; block × zone interaction, *F*_[3,6]_ = 9.033, *p* = 0.01). This pattern suggests normal learning of the cue-escape relationship but a flexible strategy shift during the probe trial, resulting in a less striking bias of search when these 15 s blocks are collapsed across the full 60 s probe trial (as manifested a non-significant main effect of zone; *F*_[1,8]_ = 0.977, *p* > 0.3; c.f. the trend-level zone bias on the day 7 probe trial in Figure 4A).

This pattern was lost in the lesioned animals (Figure 4C). There was a bias towards the goal quadrant during all 15 s subdivisions of the probe trial (RM-ANOVA: main effect of zone, *p* = 0.01), but no block × zone interaction (*F*_[3,6]_ = 0.051; *p* > 0.8). The bias towards the previously reinforced cue was present in all four blocks, though with varying degrees of statistical significance (one-tailed *t-*test for predicted effect blocks 1–4 respectively: *t*: 1.83, 2.55, 1.61, 2.53; *p* = 0.05, 0.02, 0.07, 0.02) (see Figure 4C).

## Discussion

It is now well established that the dorsal striatum has an important role in certain forms of learning. Different lines of data, including our findings in mice using the water maze task described here (Lee et al., 2008), support the idea that such dorsal striatum-dependent learning occurs in parallel with learning mediated by other brain circuits, such as a spatial learning circuit that requires the dorsal hippocampus (Mishkin and Petri, 1984; Mishkin et al., 1984; White and McDonald, 2002; McDonald et al., 2008). Under some circumstances such parallel learning systems can compete with one another (Poldrack and Packard, 2003; Daw et al., 2005), as suggested by data from both rodents (Packard et al., 1989; Schroeder et al., 2002; Lee et al., 2008) and humans (Poldrack et al., 2001).

However, the dorsal striatum is not a homogenous structure (Haber et al., 2000). Here we investigate the effect of specific DMS lesions on cued and spatial water maze learning, and find results quite different from those we previously observed after large dorsal striatal lesions. Whereas the large lesions impaired cued learning and enhanced spatial learning (Lee et al., 2008), these more restricted lesions delayed spatial learning (Figure 3) and left cued learning intact (Figure 4). Indeed, cued learning appeared enhanced after DMS lesions (Figure 4A); but a closer exploratory analysis suggests that this results from a perseverative search pattern over the course of a probe trial (Figure 4C).

Several previous studies in rats and mice have examined the differential behavioral roles of dorsolateral and DMS. In a different water maze task, Devan and White found that DMS lesions retarded learning of both spatial and cued strategies, without affecting ultimate performance (Devan et al., 1999; Devan and White, 1999). We see a similar retardation of spatial learning, though not of cued learning; this may derive from differences between our protocol and those used in these earlier studies. Devan et al. (1999) describe increased thigmotaxis after DMS lesions, which we do not observe in this experiment (Figure 3C). There are several possible reasons for this difference; it may derive simply from differences between the protocols (such as the fact that we perform a series of shaping trials, and our cued task has two cues rather than one), or a difference between mice and rats. Alternatively, it may be because our lesions did not affect the most medial, periventricular region of the striatum (Figure 1).

Restricted DMS lesions did not impair cued learning; this differs markedly from the effects of larger dorsal striatal lesions in our earlier studies (Lee et al., 2008). DMS lesions have been shown to spare cue-driven learning in a variety of contexts. For example, in a cross-maze task (Tolman et al., 1946), cue-driven “response” learning is impaired by broad manipulations of the striatum (Packard and McGaugh, 1996; Pittenger et al., 2006) but not by more restricted lesions of the DMS (Yin and Knowlton, 2004). Indeed, in this latter study, posterior DMS lesions in rats potentiated “response” learning, relative to spatial learning, similarly to what we see here in mice (in a rather different experimental paradigm).

We have also examined the involvement of the dorsolateral and DMS in instrumental conditioning in mice. We used insensitivity to reinforcer revaluation (both devaluation and inflation) to assess habitual responding and found that damage to the dorsolateral striatum preserved sensitivity to changes in outcome value following either outcome devaluation or, shown for the first time in mice, outcome inflation (Quinn et al., 2013). However, lesions to the DMS were similar to sham and did not have these effects, further distinguishing these areas’ involvement in the performance of habitual responses.

It was intriguing that performance of the cued task in DMS-lesioned animals in the day 7 probe trial tended to be *better* than that of controls (Figure 4A). As described above, a closer analysis of probe trial performance showed this effect to derive from the fact that lesioned animals tended to return to the previously reinforced cue, even late in the probe trial, while control animals switched to a broader pattern of search after failing to locate the escape platform upon early visits to the goal cue (Figures 4B, C). This suggests that intact animals could switch strategies when their initial escape attempt was unsuccessful, while DMS-lesioned animals lacked this capacity for behavioral flexibility. This finding is consistent with previous investigations of the role of a circuit including the DMS in cognitive flexibility and strategy switching, in other behavioral tasks (Ragozzino et al., 2002; Ragozzino, 2007; McDonald et al., 2008; Castane et al., 2010). To the extent that the DMS participates in behavioral flexibility, its dysfunction may contribute to perseverative patterns of behavior, such as what we see here (Figure 4C), after lesions or in neuropsychiatric disease.

We also recently described a significant deficit in prepulse inhibition (PPI) after lesions of the DMS in C57Bl/6J mice (Baldan Ramsey et al., 2011). This effect was quite specific as lesions to a neighboring central region of the dorsal striatum had no significant effect on PPI. This dissociation further emphasizes functional differentiation along the medial-lateral axis of the dorsal striatum (Yin and Knowlton, 2006). PPI is often used as an experimental measure of the ability to suppress or “gate” irrelevant information from external or internal sources and several neuropsychiatric diseases, including obsessive-compulsive disorder , are characterized with a deficit in this ability (McGhie and Chapman, 1961; Swerdlow et al., 1993; Hoenig et al., 2005). Impaired sensorimotor gating and perseveration, or the inability to inhibit a prepotent response, are both considered executive functions; these observations suggest that they share underlying neural substrates.

Studies such as this, when compared to our previously reported very different findings after larger striatal lesions, emphasize the functional heterogeneity of the striatum. Whether this heterogeneity derives from differences at the cellular or microcircuit level (Partridge et al., 2000; Yin et al., 2009) or simply from differences in connectivity with functionally distinct cortical regions (Haber et al., 2000; Gerfen, 2004) this functional heterogeneity is an important organizing principle of the basal ganglia. Furthering our understanding of the anatomical, neurochemical, and molecular facilitators of this heterogeneity and more specifically of the perseverative behavior demonstrated here may deliver better comprehension into the pathophysiological neural functions underlying behaviors in illnesses such as obsessive-compulsive disorder and related psychiatric diseases (Graybiel and Rauch, 2000; Ragozzino, 2007; Graybiel, 2008; Baldan Ramsey et al., 2011).

## Author contributions

Anni S. Lee performed experiments. Jessica M. André revised the manuscript and analyses. Christopher Pittenger supervised all experiments and analyses.

## Conflict of interest statement

The authors declare that the research was conducted in the absence of any commercial or financial relationships that could be construed as a potential conflict of interest.

## Footnotes

^1^www.jax.org
